# Biomarkers of brain injury after cardiac arrest; a statistical analysis plan from the TTM2 trial biobank investigators

**DOI:** 10.1016/j.resplu.2022.100258

**Published:** 2022-06-02

**Authors:** Marion Moseby-Knappe, Helena Levin, Kaj Blennow, Susann Ullén, Henrik Zetterberg, Gisela Lilja, Josef Dankiewicz, Janus Christian Jakobsen, Alice Lagebrant, Hans Friberg, Alistair Nichol, Kate Ainschough, Glenn M. Eastwood, Matt P. Wise, Matthew Thomas, Thomas Keeble, Alain Cariou, Christoph Leithner, Christian Rylander, Joachim Düring, Jan Bělohlávek, Anders Grejs, Ola Borgquist, Johan Undén, Maryline Simon, Vinzent Rolny, Alex Piehler, Tobias Cronberg, Niklas Nielsen

**Affiliations:** a Department of Clinical Sciences Lund, Neurology, Lund University, Skåne University Hospital, Lund, Sweden; b Department of Clinical Sciences Lund, Anaesthesiology and Intensive Care, Research and Education, Lund University, Skåne University Hospital, Lund, Sweden; c Department of Psychiatry and Neurochemistry, Institute of Neuroscience & Physiology, The Sahlgrenska Academy, University of Gothenburg, Sweden; d Clinical Neurochemistry Laboratory, Sahlgrenska University Hospital, Mölndal, Sweden; e Clinical Studies Sweden – Forum South, Skåne University Hospital, Lund, Sweden; f Department of Neurodegenerative Disease, UCL Institute of Neurology, Queen Square, London, United Kingdom; g UK Dementia Research Institute at UCL, London, United Kingdom; h Hong Kong Center for Neurodegenerative Diseases, Clear Water Bay, Hong Kong, China; i Department of Clinical Sciences Lund, Cardiology, Lund University, Skåne University Hospital, Lund, Sweden; j The Copenhagen Trial Unit, Centre for Clinical Intervention Research, Denmark; k Department of Regional Health Research, The Faculty of Health Sciences, University of Southern Denmark, Denmark; l Department of Clinical Sciences Lund, Anaesthesia and Intensive Care, Lund University, Skåne University Hospital, Malmö, Sweden; m University College Dublin, Clinical Research Centre, St Vincent's University Hospital Dublin, Ireland; n The Australian and New Zealand Intensive Care Research Centre, Monash University, Melbourne. Australia; o Intensive Care Unit, Alfred Hospital, Melbourne, Australia; p Department of Intensive Care, Austin Hospital, Melbourne, VIC, Australia; q Australian and New Zealand Intensive Care Research Centre, School of Public Health and Preventive Medicine, Monash University, Melbourne, Australia; r Adult Critical Care, University Hospital of Wales, Cardiff, United Kingdom; s Intensive Care Unit, University Hospitals, Bristol and Weston, England, United Kingdom; t Essex Cardiothoracic Centre, MSE, Basildon, Essex, United Kingdom; u MTRC, Anglia Ruskin School of Medicine, Chelmsford, Essex, United Kingdom; v Medical Intensive Care Unit, AP-HP, Cochin Hospital, Paris, France, Paris Cité University, Paris, France; w AG Emergency and Critical Care Neurology, Campus Virchow Klinikum, Department of Neurology, Charité Universitätsmedizin Berlin, Berlin, Germany; x Anaesthesiology and Intensive Care, Department of Surgical Sciences, Uppsala University, Uppsala, Sweden; y Second Department of Medicine, First Faculty of Medicine, Charles University and General University Hospital, Prague, Czech Republic; z Department of Anaesthesiology and Intensive Care Medicine, Aarhus University Hospital, Aarhus, Denmark; aa Department of Clinical Medicine, Aarhus University, Aarhus, Denmark; ab Department of Clinical Sciences Lund, Anaesthesia and Intensive Care, Lund University, Department of Cardiothoracic Surgery, Skåne University Hospital, Lund, Sweden; ac Department of Clinical Sciences Malmö, Dept. Operation and Intensive Care, Lund University, Hallands Hospital Halmstad, Halmstad, Sweden; ad Clinical Development Department, Roche Diagnostics International AG, Rotkreuz, Switzerland; ae Biostatistical Department, Roche Diagnostics International AG, Rotkreuz, Switzerland; af Department of Clinical Sciences Lund, Anaesthesia and Intensive Care, Lund University, Helsingborg Hospital, Lund, Sweden

**Keywords:** Cardiac arrest, Brain injury markers, Neurofilament light, NFL, Total-tau, glial fibrially acidic protein, GFAP, S100, Neuron specific enolase, NSE, Prognostication, outcome, biomarkers, Protocol

## Abstract

**Background:**

Several biochemical markers in blood correlate with the magnitude of brain injury and may be used to predict neurological outcome after cardiac arrest. We present a protocol for the evaluation of prognostic accuracy of brain injury markers after cardiac arrest. The aim is to define the best predictive marker and to establish clinically useful cut-off levels for routine implementation.

**Methods:**

Prospective international multicenter trial within the Targeted Hypothermia versus Targeted Normothermia after Out-of-Hospital Cardiac Arrest (TTM2) trial in collaboration with Roche Diagnostics International AG. Samples were collected 0, 24, 48, and 72 hours after randomisation (serum) and 0 and 48 hours after randomisation (plasma), and pre-analytically processed at each site before storage in a central biobank. Routine markers neuron-specific enolase (NSE) and S100B, and neurofilament light, total-tau and glial fibrillary acidic protein will be batch analysed using novel Elecsys® electrochemiluminescence immunoassays on a Cobas e601 instrument.

**Results:**

Statistical analysis will be reported according to the Standards for Reporting Diagnostic accuracy studies (STARD) and will include comparisons for prediction of good versus poor functional outcome at six months post-arrest, by modified Rankin Scale (0–3 vs. 4–6), using logistic regression models and receiver operating characteristics curves, evaluation of mortality at six months according to biomarker levels and establishment of cut-off values for prediction of poor neurological outcome at 95–100% specificities.

**Conclusions:**

This prospective trial may establish a standard methodology and clinically appropriate cut-off levels for the optimal biomarker of brain injury which predicts poor neurological outcome after cardiac arrest.

## Background

Biochemical markers from damaged neurons and glial cells can be measured quantitively in cerebrospinal fluid or blood and used as predictors of long-term neurological outcome in patients who remain comatose after out-of-hospital cardiac arrest (OHCA). Currently, only neuron-specific enolase (NSE) is recommended for this purpose by the European Resuscitation Council and the European Society of Intensive Care Medicine.^1^ Unfortunately, NSE may be falsely elevated in haemolytic samples or in the presence of tumours producing the protein.2., 3. S100B is a routinely available glial marker often used in patients with traumatic injury, but it is not ideal as a predictor of neurological outcome after OHCA.4., 5.

The novel brain injury markers total-tau, neurofilament light chain protein (NFL) and glial fibrillary acidic protein (GFAP) are more specific to the central nervous system than NSE and S100B.^6^ GFAP is an early marker of astrocytic injury and cell activation and is often used in combination with another neuronal marker after traumatic brain injury.7., 8. The neuroaxonal markers total-tau and NFL have demonstrated excellent prognostic performance after OHCA when analysed with a highly sensitive methodology and could even differentiate between degrees of brain injury.9., 10., 11. Their predictive abilities seem higher than those of NSE, S100B and GFAP, but they have never been analysed head-to-head or in combinations.2., 4., 7., 9., 10., 12. Currently, total-tau and NFL are only available on specific equipment using research grade assays. Several challenges remain to make these markers readily available on standard analytical instruments.6., 13.

Roche Diagnostics International AG has developed Elecsys®, an electrochemiluminescence immunoassay (ECLIA) for quantitative and standardised in-vitro detection of biomarkers utilising analytical platforms already commercially available.^14^ Below we describe the statistical analysis plan for our study on brain injury markers from the prospective international Targeted Hypothermia versus Targeted Normothermia after Out-of-Hospital Cardiac Arrest (TTM2) trial in collaboration with Roche Diagnostics International AG.^15^ Our study will include analysis of both clinically available and novel biochemical markers of neuronal and glial cells analysed on the same analytical platform.

The objectives of the TTM2 biobank brain injury markers are to 1) use a standardised analytical methodology to assess which routine and novel neuronal and glial brain injury marker is the best predictor of functional outcome after OHCA, and 2) establish clinically useful cut-off levels for neurological prognostication.

## Material and methods

### Patients

Between November 2017 and January 2020, the TTM2 trial prospectively randomised 1900 patients ≥ 18 years with OHCA of a presumed cardiac or with an unknown cause of arrest to hypothermia (target temperature 33 °C) or to normothermia (target temperature ≤ 37.8 °C) (clinicaltrials.gov NCT02908308).15., 16. No significant differences between the groups were found in neurological outcome or survival at six-month follow-up.^15^ Criteria for neurological prognostication and withdrawal of life-sustaining therapy have been previously published.^16^ Co-enrolment in the Targeted Therapeutic Mild Hypercapnia after Resuscitated Cardiac Arrest (TAME) trial was encouraged.^17^

### Ethics

The trial protocol was approved by the ethics committees in each participating country.^15^ Blood samples were pseudonymised before shipment and storage in the biobank. Analyses of the samples will be performed without any personal identifiers and published research results will only be on a group-level and not be traceable back to any individual participant.

### Outcomes

The prognostic accuracy of biomarkers will be evaluated as the ability to predict neurological disability as functional outcome at six months post-arrest by a binary modified Rankin Scale (mRS), dichotomised into “good” (mRS) 0–3) or “poor” (mRS 4–6) outcome as previously described.15., 18.

### Biochemical analysis of brain injury markers

This protocol describes the analyses of brain injury markers from the twenty-five TTM2 sites participating in the biobank by Roche Diagnostics International AG.15., 16. 7/25 sites co-enrolled patients to the TAME trial.^15^ Blood was sampled prospectively and processed at each participating site, aliquoted, and frozen to − 80 °C before shipment to the Integrated BioBank of Luxembourg. Serum was stored from the timepoint of randomisation (0 hours) and from 24, 48, and 72 hours after randomisation, whilst plasma was stored from 0 and 48 hours after randomisation. The biobank instructions for collection of samples are detailed in supplement 1. Samples will be analysed with research prototype [NSE, S100] and novel prototype [NFL, total-tau, GFAP] Elecsys® electrochemiluminescence immunoassays (ECLIA) for quantitative in-vitro detection of biomarkers on a Cobas e601 System at the Roche Research Lab Facilities in Penzberg (Germany). Table 1 displays the limits of quantification. The presence of haemolysis will be determined in the serum and plasma aliquots used to measure biomarker concentrations with a Roche haemolysis index at 600 and 570 nm.^19^ NSE levels may be routinely available for neuroprognostication at some sites and results of the local analysis will be collected separately.

• The primary analysis of brain injury markers will utilize serum samples at 0, 24, 48, and 72 h post-randomisation for measurement of NSE, S100B, NFL and GFAP.
• In a second phase, plasma samples available at 0 and 48 h will be utilized for analyses of total-tau, GFAP and NFL.

**Table 1:** Limits of quantification for brain injury markers for Elecsys®.

**Brain injury marker**	**Lower limit of quantification**	**Range**
NSE	0.05 ng/mL	0.05–370 ng/mL
S100B	0.005 µg/L	0.005–39 µg/L
NFL	0.21 pg/mL	0.21–1959 pg/mL
GFAP	0.004 ng/mL	0.004–200 ng/mL
Total tau	0.18 pg/mL	0.18–3895 pg/mL

The lower limit of quantification and range. Samples with high concentrations will be diluted to determine exact concentrations.

### Statistical analysis of brain injury markers

Results from the biochemical analyses will first become available to the TTM2 biobank working group after this statistical analysis plan has been submitted for publication. The statistical analysis will be performed parallel and independently by the TTM2 biobank working group and by the medical statisticians from Roche Diagnostics International AG. Results of the statistical analyses will be compared and added as supplements to any publication. All analyses will be published regardless of the results. Reporting will follow the Standards for Reporting Diagnostic accuracy studies (STARD) and Transparent Reporting of a multivariable prediction model for Individual Prognosis Or Diagnosis (TRIPOD).20., 21.

We plan to perform our analyses as follows; I) description of the population, haemolysis, and effect of interventions on the prognostic performance of biomarkers; II) main aims; prediction of functional outcome, determination of cut-off values, and mortality and III) exploratory analyses. Biomarker levels may be analysed transformed into a logarithmic scale if appropriate.

## I) Description of the population, haemolysis, and effect of interventions on the prognostic performance of biomarkers

### Population

**Fig. 1:**
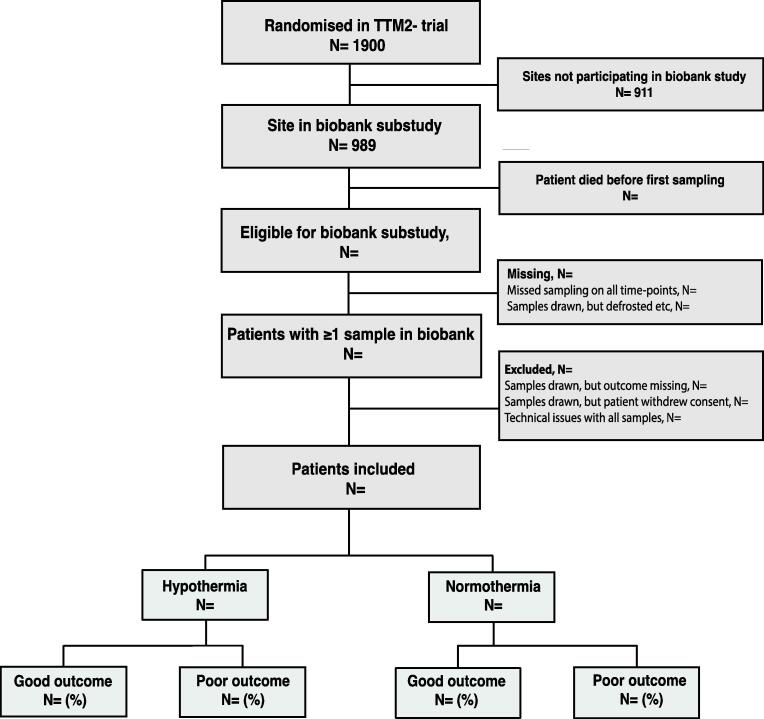
**Example of patient flow-chart.** The flow-chart will be reported in accordance with the Standards for Reporting of Diagnostic Accuracy Studies (STARD) and the Transparent Reporting of a multivariable prediction model for Individual Prognosis Or Diagnosis (TRIPOD).20., 21.

Patients were consecutively included at participating sites. We will describe reasons for exclusion in a flowchart (Fig. 1) and the number of available samples at each time point (eTable 1). We will present baseline characteristics of included and excluded patients, since any differences might influence the interpretation of the results (eTable 2). The main analysis will include all patients with data available at each timepoint.

### Correlation with clinical characteristics

Biomarkers will be examined for their association with the TTM2 trial design variables; age (continuous), sex (female/male), time to ROSC (continuous), shockable initial rhythm (shockable versus non-shockable) and circulatory shock on admission (present versus not present). The association between the design variables and biomarker levels will be described using Spearman’s rho for continuous variables, and by comparing the biomarker levels between binary variables using the Mann-Whitney-Wilcoxon-Test (eTable 3).

### Haemolysis

If biomarker levels on a group level are significantly higher in samples ≥ 500 mg/l haemolysis than in samples < 500 mg/l haemolysis, we will consider such samples pre-analytically confounded and exclude them from further analysis of that specific marker (eFig. 1). Only NSE is present in erythrocytes and has previously demonstrated elevated blood levels in haemolytic samples.^2^

### Effect of interventions on the prognostic performance of biomarkers

We will describe and graphically inspect distributions of biomarker levels between the temperature groups for good and poor functional outcome (eFig. 2). In an interaction model we will examine if the effect of the biomarker is similar in the temperature groups (eTable 4A). If the interaction effect is not statistically significant, we will pool data when analysing prognostic accuracies as described in II). If required, we will present prognostic accuracies separately for each intervention group. Due to the co-enrolment with the TAME trial, we will also present interaction models for patients randomised to mild hypercapnia (PaCO2, 50–55 mmHg) or standard care (PaCO2, 35–45 mmHg) (eTable 4B).

## II) Prediction of functional outcome, determination of cut-off values, and mortality

### Prediction of functional outcome

**Fig. 2:**
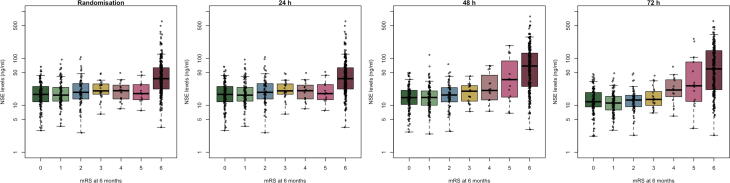
**Example biomarker levels according to functional outcome (mRS).** The boxplots with scatter will be made separately for each biomarker and timepoint and will include serum concentrations of all patients, irrespective of TTM allocation at each level of the modified Rankin Scale (mRS) at six months post cardiac arrest. *Figure example based on data from the TTM trial.*2., 9., 24.

Firstly, we will display concentrations of biomarkers in relation to functional outcome according to separate levels of the mRS at six months (Fig. 2).

**Fig. 3:**
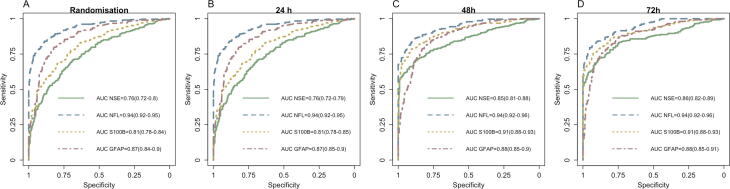
**Example figure for ROC analysis for overall prognostic accuracies.** This ROC analysis is the main analysis and will include all available data from patients with biomarker data and functional outcome (good versus poor) at six months. Using paired ROC curves (DeLong) we will examine whether the difference in AUROC between the two best markers at each time-point is statistically significant. We will correct for multiplicity of the time-points (n=4 comparisons for serum samples and n= 2 for plasma), where p< 0.125 and p<0.025 will be considered statistically significant. *Figure example based on data from the TTM trial.*2., 4., 7., 10., 12., 24.

Secondly, we will examine the prognostic accuracy for binary functional outcome (good versus poor). Using logistic regression models, we aim to analyse whether biomarkers are useful additions to the TTM2 design variables for prediction of functional outcome (Table 2). The overall precision of the biomarkers for prediction of functional outcome will also be presented as area under the receiver operating characteristics curve (AUROC) with 95% confidence intervals (Fig. 3). P-values will be calculated based on a test of difference in AUROC using the method of DeLong. For the main logistic regression models on overall AUROC for prediction of functional outcome, we aim to compare the prognostic accuracies of the two brain injury markers with the highest AUROC at each timepoint to each other. P-values will then be adjusted for multiplicity based on the number of timepoints of sample collection (n = 4 for serum and n = 2 for plasma), where p < 0.0125 and p < 0.025 will be considered statistically significant, respectively. We will also report partial AUROC at high (95–100%) specificities in all patients (eTable 5). In a subgroup analysis, we will examine overall prognostic accuracies in unconscious patients alive but not awake who would be eligible for neurological prognostication (eFig. 3). We will examine whether the AUROC of the Δ change in biomarker levels between timepoints can be used for prediction of functional outcome (eTable 6).

**Table 2:** Example of logistic regression models adding biomarkers to clinical information for prediction of functional neurological outcome.

**Timepoint**	**Model predictors**	β **biomarker** **(95% CI)**	**AIC**	**AUC**
Randomisation	Clinical variables Biomarker only Clinical and biomarker	**NA**		
24 h	Clinical variables Biomarker only Clinical and biomarker	**NA**		
48 h	Clinical variables Biomarker only Clinical and biomarker	**NA**		
72 h	Clinical variables Biomarker only Clinical and biomarker	**NA**		

**Examples of graphical presentation of results**

Logistic regression models will be reported separately for each biomarker for prediction of good versus poor functional outcome at 6 months’ follow-up. “Clinical information” included age, sex, time to ROSC, TTM allocation, shock on admission and whether initial rhythm on ECG was shockable. For each model, we determine coefficients for the biomarkers (**β =** increase in log odds for poor functional outcome for each log10 unit increase in serum concentration with 95% confidence intervals), Akaike Information criterion (AIC, measure of model fit; smallest is preferable) and Area Under the Receiver Operating Characteristics curve (AUC). For biomarkers where a logarithmic-transformation of serum levels is not appropriate, other transformations or categorisation will be considered. We will calculate p-values for comparing if difference in AUC between the clinical information and the model including biomarker plus clinical information is statistically significant.

The conclusion on which brain injury marker is the optimal predictor of functional outcome marker will be based on the comparison of AUROC (overall and partial) for single biomarkers.

### Cut-off values at high specificities

We will present cut-offs and sensitivities at high specificities (95–100%) and as the Youden-index for each timepoint for good versus poor functional outcome at six months (eTable 7). Sensitivities and specificities at all cut-offs will be determined by an out-of-sample cross-validation procedure. In each iteration (suggested n = 10.000), 70% of participants will be chosen, as a training set and the remaining 30% of participants as a test set. Cut-offs will be determined in the training set and these cut-offs will then be evaluated for sensitivity and specificity in the test set as recommended for external validation studies.^21^ None of the sites and patients used to train the models will be used when testing the models. The reported sensitivities and specificities will be the mean results among the 10,000 test sets. The 95% confidence intervals will be calculated with Wilson’s method.^22^

### Survival analysis

**Fig. 4:**
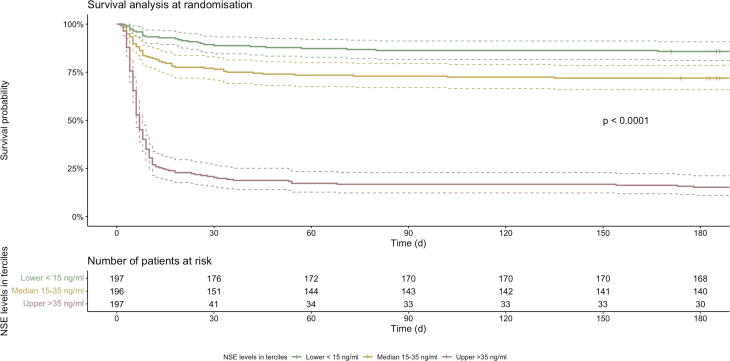
**Example figure survival analysis**. Kaplan-Meier curves for all-cause mortality up till 180 days post-arrest will be created by splitting the biomarkers into three equally large groups (tertiles with low, intermediate, and high levels of biomarkers) and using these factorial variables as a predictor for age-adjusted survival with 95% confidence intervals. Biomarker levels may be analysed transformed into a logarithmic scale if appropriate. P-values will be calculated with a log-rank test. *Figure example based on data from the TTM trial.*2., 9., 12., 24.

We will examine levels of brain injury markers according to the all-cause mortality and according to the presumed cause of death (neurological versus non-neurological according to treating physician) until 180 days post-arrest. Kaplan-Meier curves will be created for each time-point by splitting the biomarkers into three equally large groups (tertiles with low, intermediate, and high levels of biomarkers) and using these factorial variables as a predictor for age adjusted survival with 95% confidence intervals (Fig. 4). P-values will be calculated with a log-rank test.

## III) Exploratory analyses

• Validation of the currently recommended NSE cut-off for prediction of poor functional outcome ≥ 60 ng/mL at 48 and/or 72 hours post-arrest using both results from local analysis and from the Elecsys®^1^
• Examination of whether combinations of brain injury markers improve prediction of functional outcome
• Correlation of awakening at any time-point to brain injury marker levels
• Correlation of biomarker levels with other neuroprognostic methods (neurophysiology, neuroimaging and clinical neurological findings)
• Validation of prognostic accuracies for “normal concentrations” and twice normal concentrations in patients with “indeterminate outcome”1., 23.
• Correlation of brain injury markers with cognitive function by the MoCA (Montreal Cognitive Assessment) and SDMT (Symbol Digit Modalities Test)

### Publication

A first manuscript will include analyses of NSE, S100B, NFL and GFAP in *serum*. A second manuscript will include analyses of NFL, GFAP and total-tau in *plasma*. Additional exploratory analyses will be performed subsequently and may be presented in separate manuscripts.

Example figures and tables intended to be produced in the primary analysis are shown in Table 2, Fig. 1, Fig. 2, Fig. 3, Fig. 4, eTables 1–7 and eFig. 1–3. The choice of figures and tables included in the final manuscripts rather than the supplement will be based on the results (*i.e.*, depending on which biomarker shows superior results).

## Discussion

In a prospective international multicentre trial, we will use the Elecsys® methodology to compare the neuroprognostic ability of routine and novel brain injury markers after cardiac arrest. We aim to examine which neuronal and glial markers best predict brain injury and functional outcome and to establish clinically useful cut-off levels for neurological prognostication. The clinical use of novel brain injury markers are currently limited by a lack of validation, research grade assays and prolonged time for obtaining results from external laboratories.^6^ Biochemical analysis on platforms in clinical use could make novel markers more easily available.

Within the first TTM trial, both NFL and total-tau were examined using ultrasensitive novel assays, whilst NSE, S100B and GFAP were analysed with standard methods.2., 4., 10., 11. Another novel neuronal marker, ubiquitin-carboxy hydrolase L1, was not as predictive of neurological outcome as the neuroaxonal markers and is therefore not included in the present study.^7^ It is unclear to which extent the analytical platform affects the prognostic accuracy. The current trial may help clarify this by using a standardised approach to analysis for all markers, both in serum and plasma.

We previously found that prognostic accuracies of biomarkers were not significantly influenced by target temperature and will therefore perform analyses on pooled data where we find no significant interaction effect between intervention groups.2., 7., 9., 10. We do not expect mild hypercapnia to influence biomarker levels, but will assess if there is a significant interaction effect for the generalisability of our results.^17^ If we do identify differences between the intervention groups, we plan to present results separately. Clinically it would be preferable to have identical cut-offs for outcome prediction regardless of the approach to intensive care management. Nonetheless, the astroglial markers S100B and GFAP previously demonstrated higher concentrations in hypothermia despite similar prognostic accuracies, which may require that prognostic accuracies for these markers are reported separately for each intervention group.4., 7.

Our statistical analyses will also include studies of biomarker dynamics for prediction of outcome. This gives us the opportunity to examine whether the increase or decrease of biomarker levels between timepoints is more relevant for outcome prediction than the quantitative levels of biomarkers themselves. Evaluation of dynamics could help distinguish patients with elevated biomarker levels due to pre-existing neurological comorbidities or haemolysis from patients with post-arrest brain injury. Within the TTM2 trial we prospectively collected samples from the time-point of randomisation, which is earlier than the first collected samples within the TTM trial biobank at 24 hours post-arrest.^2^ Combining information gained from both neuronal and glial markers over time could help us gain further understanding of the pathophysiology of hypoxic-ischaemic brain injury.

## Strengths and limitations

Strengths of the TTM2 trial include the prospective international multicenter design, randomisation to TTM stratified by site, a standardised level of sedation, conservative criteria for neurological prognostication and withdrawal of life-sustaining therapy following a detailed protocol, and a structured long-term follow up of survivors 15., 16. Analyses will be performed in batch using a standardised approach on the same analytical platform for all biomarkers. Since analysis will be performed after trial completion, results were not available upon clinical decision-making. Statistical analyses will be performed both by the TTM2 biobank working group and by medical statisticians by Roche Diagnostics International AG. Results will be published regardless of the results.

Limitations include that NSE analyses (performed locally at a participating site) were part of the recommended neuroprognostication procedure of the TTM2 trial and thus available for treating physicians. To our knowledge, NFL levels may have been available as research grade assays in one participating center. Also, information obtained from other methods used (neurophysiology, imaging) when predicting neurological outcome may bias final outcome and indirectly this study. This is, however, a commonly reported limitation for neuroprognostic studies and the protocol was conservative compared to current guidelines.^1^ The participation in the TTM2 biobank was limited to specific sites, yet we do not consider this to be a selection bias as all patients at the participating sites were eligible for inclusion. The TTM2 trial included adult patients with a presumed cardiac or unknown cause of out-of-hospital cardiac arrest. Our results may therefore require further validation in other patient populations.

## Conclusions

We present the design of a large prospective trial examining the prognostic accuracy of the most promising brain injury markers following cardiac arrest. Our results may help establish a standard methodology and appropriate cut-off levels for prognostication of neurological outcome after cardiac arrest.

## Funding

The TTM2 trial is supported by independent research grants from nonprofit or governmental agencies (the Swedish Research Council [Vetenskapsrådet], Swedish Heart–Lung Foundation, Stig and Ragna Gorthon Foundation, Knutsson Foundation, Laerdal Foundation, Hans-Gabriel and Alice Trolle-Wachtmeister Foundation for Medical Research, and Regional Research Support in Region Skåne) and by governmental funding of clinical research within the Swedish National Health Service.^15^ Biochemical markers will be analysed free of charge by Roche Diagnostics International AG, Rotkreuz, Switzerland.

KB is supported by the Swedish Research Council (#2017-00915), the Alzheimer Drug Discovery Foundation (ADDF), USA (#RDAPB-201809-2016615), the Swedish Alzheimer Foundation (#AF-930351, #AF-939721 and #AF-968270), Hjärnfonden, Sweden (#FO2017-0243 and #ALZ2022-0006), the Swedish state under the agreement between the Swedish government and the County Councils, the ALF-agreement (#ALFGBG-715986 and #ALFGBG-965240), the European Union Joint Program for Neurodegenerative Disorders (JPND2019-466-236), the National Institute of Health (NIH), USA, (grant #1R01AG068398-01), and the Alzheimer’s Association 2021 Zenith Award (ZEN-21-848495).

HZ is a Wallenberg Scholar supported by grants from the Swedish Research Council (#2018-02532), the European Research Council (#681712 and #101053962), Swedish State Support for Clinical Research (#ALFGBG-71320), the Alzheimer Drug Discovery Foundation (ADDF), USA (#201809-2016862), the AD Strategic Fund and the Alzheimer's Association (#ADSF-21-831376-C, #ADSF-21-831381-C and #ADSF-21-831377-C), the Olav Thon Foundation, the Erling-Persson Family Foundation, Stiftelsen för Gamla Tjänarinnor, Hjärnfonden, Sweden (#FO2019-0228), the European Union’s Horizon 2020 research and innovation programme under the Marie Skłodowska-Curie grant agreement No 860,197 (MIRIADE), the European Union Joint Programme – Neurodegenerative Disease Research (JPND2021-00694), and the UK Dementia Research Institute at UCL (UKDRI-1003).

## Role of the funders

Roche Diagnostics International AG, Rotkreuz, Switzerland will analyse the serum and plasma samples free of charge and a formal agreement has been settled between the TTM2 trial sponsor (Region Skåne, Helsingborg hospital, Sweden) and Roche Diagnostics. Prior to analysing samples, the current statistical analysis plan was defined in collaboration between the TTM2 trial investigators, medical statisticians from both Clinical Studies Sweden and Roche Diagnostics International AG and by scientists from Roche Diagnostics International AG. Statistical analyses will be performed twice (parallel and independently) according to the statistical analysis plan by investigators from the TTM2 trial in collaboration with a medical statistician from Clinical Studies Sweden and by a medical statistician from Roche Diagnostics International AG. Any discrepancies in the analyses will be discussed and solved by consensus and reported in the publications. The manuscript will be written by investigators from the TTM2 trial with the representatives of Roche Diagnostics International AG as co-authors. The results of the analyses will be published as described regardless of the results.

The remaining funding organisations had no role in the design and conduct of the study; collection, management, analysis, and interpretation of the data; preparation, review, or approval of the manuscript; and decision to submit the manuscript for publication.

## Disclosures

MS, VR and AP are employed by Roche Diagnostics International AG. KB has served as a consultant, at advisory boards, or at data monitoring committees for Abcam, Axon, BioArctic, Biogen, JOMDD/Shimadzu, Julius Clinical, Lilly, MagQu, Novartis, Ono Pharma, Pharmatrophix, Prothena, Roche Diagnostics International, and Siemens Healthineers, and is a co-founder of Brain Biomarker Solutions in Gothenburg AB (BBS), which is a part of the GU Ventures Incubator Program, outside the work presented in this paper. HZ has served at scientific advisory boards and/or as a consultant for Abbvie, Alector, Annexon, Artery Therapeutics, AZTherapies, CogRx, Denali, Eisai, Nervgen, Novo Nordisk, Pinteon Therapeutics, Red Abbey Labs, Passage Bio, Roche, Samumed, Siemens Healthineers, Triplet Therapeutics, and Wave, has given lectures in symposia sponsored by Cellectricon, Fujirebio, Alzecure, Biogen, and Roche, and is a co-founder of Brain Biomarker Solutions in Gothenburg AB (BBS), which is a part of the GU Ventures Incubator Program (outside submitted work). AG has received honoraria from Bard Medical for lectures at educational meetings. TK holds a research grant from Zoll Medical, and speaker fees from Bard Medical - both companies with technology in temperature management. MPW has received honoraria from Gilead and Fisher & Paykel for lectures at educational meetings. No other potential conflict of interest relevant to this article was reported.
