# Supplementary material for: Biomarkers of brain injury after cardiac arrest; a statistical analysis plan from the TTM2 trial biobank investigators
Source: Resusc Plus. 2022 Jun 2;10:100258. doi: 10.1016/j.resplu.2022.100258 (PMC9168690; doi:10.1016/j.resplu.2022.100258)
Supplement: Supplementary data 1 [file mmc1.pdf]

Online supplementary

Biomarkers of brain injury after cardiac arrest; a statistical analysis plan from the TTM2 trial biobank investigators

Marion Moseby-Knappe, Helena Levin, Kaj Blennow, Susann Ullén, Henrik Zetterberg, Gisela Lilja, Josef Dankiewicz, Janus Christian Jakobsen, Alice Lagebrant, Hans Friberg, Alistair Nichol, Kate Ainschough, Glenn M. Eastwood, Matt P. Wise, Matthew Thomas, Thomas Keeble, Alain Cariou, Christoph Leithner, Christian Rylander, Joachim Düring, Jan Bělohlávek, Anders Grejs, Ola Borgquist, Johan Undén, Maryline Simon, Vincent Rolny, Alex Piehler, Tobias Cronberg* and Niklas Nielsen*

*Authors contributed equally

Corresponding author:
Marion Moseby-Knappe, MD, PhD
Clinical Sciences Lund
Department of Neurology
Skåne University Hospital
22185 Lund
Sweden

Table of Contents

eTable 1. Example serum samples available for analysis 3
eTable 2. Example clinical characteristics and baseline variables 4
eFig. 1. Example haemolysis 5
eTable 3. Example association between serum concentrations and design variables 6
eFig. 2. Example figure boxplot for biomarker levels within temperature groups 7
eTable 4A. Example interaction model for the effect of targeted temperature management on the prognostic performance of biomarkers 8
eTable 4B. Example interaction model for the effect of mild hypercapnia on the prognostic performance of biomarkers 8
eTable 5. Example prognostic accuracies for single markers (overall and partial AUROC) 9
eFig. 3. Example ROC analysis for overall prognostic accuracies in unconscious patients only 10
eTable 6. Example ROC analysis for change in biomarker levels between time-points 11
eTable 7. Example cut-off values and sensitivities at high specificities 12
Supplement 1. Version 1.1 of the biobank instructions for collection of samples 13

eTable 1. Example serum samples available for analysis

		NSE	S100B	NFL	GFAP
**Randomisation**					
Patients included in biobank, N=					
	Samples available	N (%)	N (%)	N (%)	N (%)
	Missed sampling	N (%)	N (%)	N (%)	N (%)
	Preanalytical failure	N (%)	N (%)	N (%)	N (%)
	Analytical failure				
	Haemolysis ≥500 mg/l	N (%)	N (%)	N (%)	N (%)
**24 h**					
Patients included in biobank, N=					
	Samples available	N (%)	N (%)	N (%)	N (%)
	Missed sampling due to death or moribund patient	N (%)	N (%)	N (%)	N (%)
	Missed sampling other	N (%)	N (%)	N (%)	N (%)
	Preanalytical failure	N (%)	N (%)	N (%)	N (%)
	Analytical failure	N (%)	N (%)	N (%)	N (%)
	Haemolysis ≥500 mg/l	N (%)	N (%)	N (%)	N (%)
**48 h**					
Patients included in biobank, N=					
	Samples available	N (%)	N (%)	N (%)	N (%)
	Missed sampling due to death or moribund patient	N (%)	N (%)	N (%)	N (%)
	Missed sampling other	N (%)	N (%)	N (%)	N (%)
	Preanalytical failure	N (%)	N (%)	N (%)	N (%)
	Analytical failure	N (%)	N (%)	N (%)	N (%)
	Haemolysis ≥500 mg/l	N (%)	N (%)	N (%)	N (%)
**72 h**					
Patients included in biobank, N=					
	Samples available	N (%)	N (%)	N (%)	N (%)
	Missed sampling due to death or moribund patient	N (%)	N (%)	N (%)	N (%)
	Missed sampling other	N (%)	N (%)	N (%)	N (%)
	Preanalytical failure	N (%)	N (%)	N (%)	N (%)
	Analytical failure	N (%)	N (%)	N (%)	N (%)
	Haemolysis ≥500 mg/l	N (%)	N (%)	N (%)	N (%)

For plasma samples this table will be limited to the brain injury markers NFL, GFAP and tau at 0 hours and 48 hours post-randomisation. Preanalytical failure includes samples drawn, but not in biobank due to errors with labelling, samples lost at lab etc.

eTable 2. Example clinical characteristics and baseline variables

	**Included N=**	**Excluded N=**
**Demographic characteristics**		
Age (years)	Mean (SD)	Mean (SD)
Male	N (%)	N (%)
**Characteristics of cardiac arrest**		
Minutes from CA to ROSC	Median (IQR)	Median (IQR)
First monitored rhythm on ECG shockable	N (%)	N (%)
Bystander witnessed CA	N (%)	N (%)
Bystander CPR performed	N (%)	N (%)
Cardiac arrest at the place of residence	N (%)	N (%)
**Clinical characteristics on hospital admission**		
Corneal reflexes bilaterally absent on hospital admission	N (%)	N (%)
Pupillary reflexes bilaterally absent on hospital admission	N (%)	N (%)
Tympanic temperature on admission	Mean (SD)	Mean (SD)
ST-segment elevation myocardial infarction	N (%)	N (%)
Arterial lactate level on admission mmol/L	Mean (SD)	Mean (SD)
Circulatory shock on admission	N (%)	N (%)
Randomised to hypothermia	N (%)	N (%)
**Medical history**		
Hypertension	N (%)	N (%)
Dementia	N (%)	N (%)
Cerebrovascular disease	N (%)	N (%)
Hemiplegia	N (%)	N (%)
Diabetes	N (%)	N (%)
Myocardial infarction	N (%)	N (%)
Heart failure NYHA III or IV	N (%)	N (%)
Renal failure	N (%)	N (%)
Moderate or severe liver failure	N (%)	N (%)
Charlson comorbidity index	Median (IQR)	Median (IQR)
**Functional outcome after 180 days**		
Good outcome (mRS 0-3)	N (%)	N (%)
Poor outcome (mRS 4-6)	N (%)	N (%)
**Structured assessment of neurological outcome at 180 days**		
mRS 0	N (%)	N (%)
mRS 1	N (%)	N (%)
mRS 2	N (%)	N (%)
mRS 3	N (%)	N (%)
mRS 4	N (%)	N (%)
mRS 5	N (%)	N (%)
mRS 6	N (%)	N (%)
**Withdrawal of life-sustaining therapy (WLST)**		
WLST performed	N (%)	N (%)
WLST neurological reason only	N (%)	N (%)

Data will be presented in numbers (percentages) for categorical variables, and in mean (standard deviation) or median (interquartile range) as appropriate for continuous variables.

eFig. 1. Example haemolysis

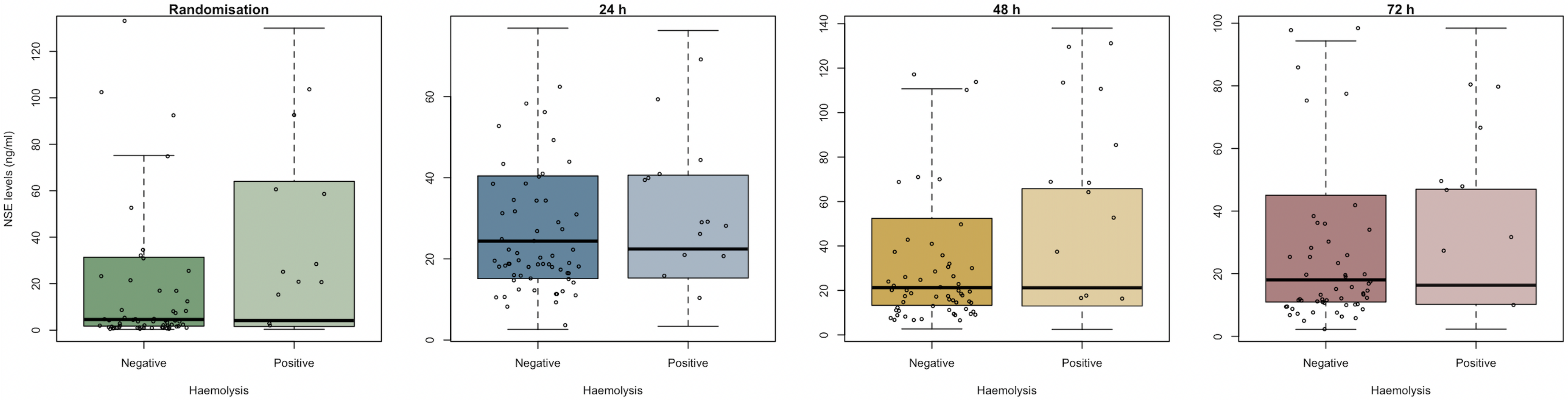

For each biomarker we will examine whether a Roche haemolysis index measured at 600 and 570 nm ≥500 mg/l haemolysis is associated with higher levels the biomarker. In case there is a significant difference (Mann-Whitney-Wilcoxon Test), samples with a haemolysis index above cut-off will be removed for those biomarkers where haemolysis may affect results. (Figure example based on data from the TTM trial).

eTable 3. Example association between serum concentrations and design variables

	NSE	S100B	NFL	GFAP
Age per year				
Male sex				
Time to ROSC per minute				
Circulatory shock on admission				
Initial rhythm shockable				

We will examine the correlation between the pre-specified design variables and each biomarker using Spearman's rho for continuous variables, and by comparing median biomarker levels between binary variables using the Mann-Whitney-Wilcoxon-Test.

eFig. 2. Example figure boxplot for biomarker levels within temperature groups

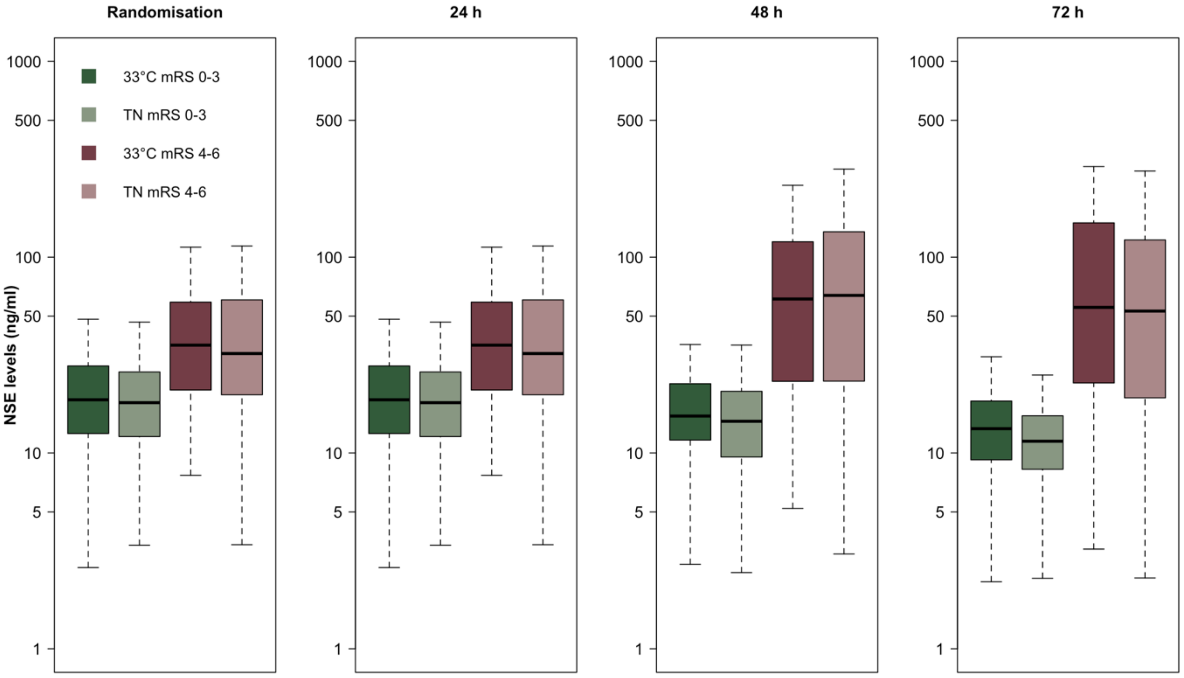

Boxplots will be presented separately for the serum levels of each biomarker according to TTM group (hypothermia (33°C) and normothermia (TN) and functional neurological outcome at 0, 24, 48, and 72 hours post-randomisation. *(Example figure based on data from the TTM trial).*

eTable 4A. Example interaction model for the effect of targeted temperature management on the prognostic performance of biomarkers

**Timepoint**	**Model predictors**	**β biomarker (95% CI)**	**p-value Interaction effect**
Randomisation	Hypothermia<br>Normothermia		
24 h	Hypothermia<br>Normothermia		
48 h	Hypothermia<br>Normothermia		
72 h	Hypothermia<br>Normothermia		

We will examine the effect of the temperature intervention on the coefficients for the biomarkers (**β**=increase in log odds for poor functional outcome for each log10 unit increase in serum concentration with 95% confidence intervals). If the interaction effect of target temperature is non-significant, we will pool data of all patients, regardless of temperature intervention, for calculation of prognostic accuracies of that biomarker.

eTable 5B. Example interaction model for the effect of mild hypercapnia on the prognostic performance of biomarkers

**Timepoint**	**Model predictors**	**β biomarker (95% CI)**	**p-value Interaction effect**
Randomisation	Standard care<br>Hypercapnia		
24 h	Standard care<br>Hypercapnia		
48 h	Standard care<br>Hypercapnia		
72 h	Standard care<br>Hypercapnia		

We will examine the effect of mild hypercapnia ($PaCO_2$, 50–55 mmHg) versus standard care ($PaCO_2$, 35–45 mmHg) on the coefficients for the biomarkers (**β**=increase in log odds for poor functional outcome for each log10 unit increase in serum concentration with 95% confidence intervals). If the interaction effect of mild hypercapnia is non-significant, we will pool data of all patients for calculation of prognostic accuracies of that biomarker.

eTable 6. Example prognostic accuracies for single markers (overall and partial AUROC)

**Timepoint**	**Brain injury marker**	**Overall AUROC (95% CI)**	**AIC**	**Partial AUROC spec $_{95\text{-}100\%}$ (95% CI)**	**AIC**
Randomisation	NSE<br>S100B<br>NFL<br>GFAP				
24 h	NSE<br>S100B<br>NFL<br>GFAP				
48 h	NSE<br>S100B<br>NFL<br>GFAP				
72 h	NSE<br>S100B<br>NFL<br>GFAP				

Overall AUROC includes prognostic accuracies for differentiating between good and poor functional outcome at all specificities using all available data. Partial AUROC includes AUROC at high specificities (95-100%) x 20, so that the maximum AUROC for each model is still 1. AIC= Akaike Information Criterion as a measure of model-fit.

eFig. 3. Example ROC analysis for overall prognostic accuracies in unconscious patients only

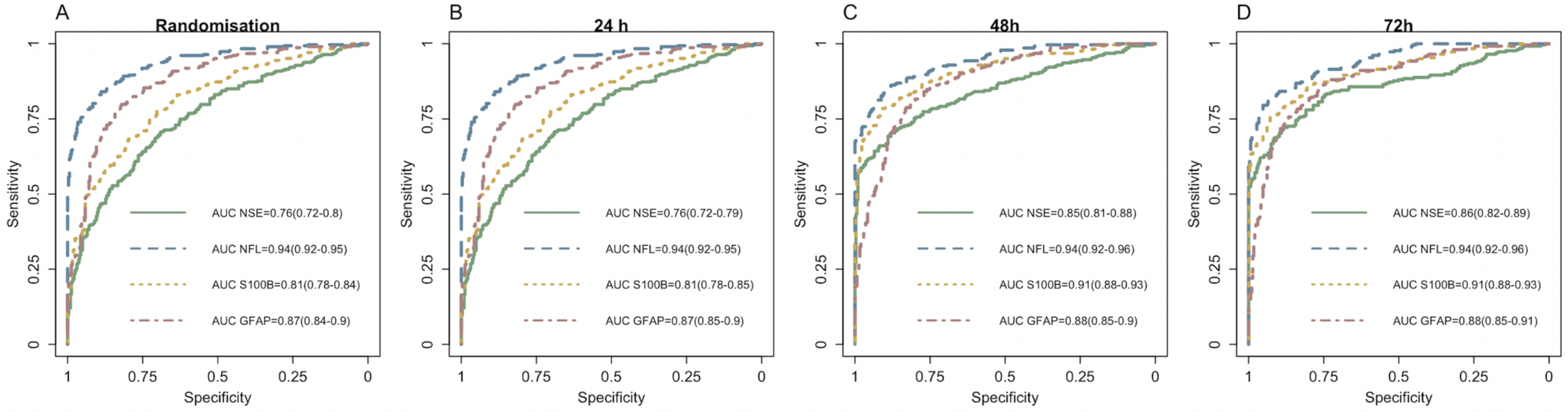

In this exploratory analysis, at each timepoint we will exclude patients who would not be subject to neurological prognostication (awake and obeying commands or moribund at the timepoint of sample collection). Functional outcome will be defined as good or poor at six months. (Figure example based on data from the TTM trial).

eTable 7. Example ROC analysis for change in biomarker levels between time-points

	**Δ 0h vs 24 h**	**Δ 0h vs 48 h**	**Δ 0 h vs 72 h**	**Δ 24 h vs 48 h**	**Δ 24 h vs 72 h**	**Δ 48 h vs 72 h**
**AUROC**						

The Δ (delta) change in blood levels between two time-points will be used to calculate the overall AUROC for prediction of good versus poor functional neurological outcome at six months post-arrest. 0 hour indicates the timepoint of randomisation.

eTable 8. Example cut-off values and sensitivities at high specificities

			**Training set**		**Test set**	
**Timepoint**	**N=**	**Cut-off**	**Specificity (95% CI)**	**Sensitivity (95% CI)**	**Specificity (95% CI)**	**Sensitivity (95% CI)**
Randomisation			1.00			
			0.99			
			0.98			
			0.97			
			0.96			
			0.95			
			Youden			
24 h			1.00			
			0.99			
			0.98			
			0.97			
			0.96			
			0.95			
			Youden			
48 h			1.00			
			0.99			
			0.98			
			0.97			
			0.96			
			0.95			
			Youden			
72 h			1.00			
			0.99			
			0.98			
			0.97			
			0.96			
			0.95			
			Youden			

The following table will be presented separately for each biomarker with cut-offs and sensitivities with 95% confidence intervals at 95% -100% specificities and at Youden index for good versus poor functional outcome at six months. Sensitivities and specificities at all cut-offs will be determined by an out-of-sample cross-validation procedure. In each iteration (suggested n = 10.000), 70% of participants will be chosen as a training set and the remaining 30% participants as a test set. Cut-offs will be determined in the training set and these cut-offs will then be evaluated for sensitivity and specificity in the test set. None of the sites and patients used to train the models will be used when testing the models. The reported sensitivities and specificities will be the mean results among the 10.000 test sets. The 95% confidence intervals will be calculated with Wilson´s method.

TTM2 - TAME

Blood Collection and Processing Instructions for Biobank Samples in the TTM2 and TAME Trials

Version 1.1
February 26th, 2018

TABLE OF CONTENTS

1. THE TTM2 AND TAME TRIALS **3**
2. BEDSIDE SAMPLE COLLECTION PROCESS **3**
   - 2.1 Blood collection 3
   - 2.2 Sample collection kits 3
   - 2.3 Sample collection forms 4
3. SAMPLE PREPARATION AND PROCESSING AT THE SITE LABORATORY **4**
   - 3.1 Materials for sample processing 4
   - 3.2 How to apply the labels 4
   - 3.3 Sample processing instruction 5
   - 3.4 Sample storage log 6
4. SHIPMENT **6**
5. CONTACT **6**

APPENDIX A: SAMPLE COLLECTION FORM

APPENDIX B: SAMPLE STORAGE LOG

1. THE TTM2 AND TAME TRIALS

The TTM2 (Targeted Temperature Management after Cardiac Arrest 2) and TAME (Targeted Therapeutic Mild Hypercapnia After Resuscitated Cardiac Arrest) trials will collaborate and send samples to a shared biobank located at the Integrated Biobank of Luxembourg (IBBL). Several centres will participate in both trials, but for those patients who only participate in one of the trials the samples will be used in accordance with the consent, by either TTM2 or TAME.

2. BEDSIDE SAMPLE COLLECTION PROCESS

2.1. Blood Collection

Blood samples will be collected at the site (often the ICU, but could be the Emergency Department or a Ward) from an existing central venous or arterial access device according to the table below.

Blood samples should be drawn at 0h, 24h, 48h, and 72h after randomisation. If laboratory processing according to the instructions is not possible outside "office hours", samples may be drawn +/- 6 hours from the designated time point. **Efforts should be made to obtain blood samples as close as possible to the ideal collection time points.**

Time point	Blood volume	Collection tube	Processing
0 h*	6 ml<br>6 ml<br>2,5 ml	Clot activator tube<br>EDTA-tube<br>PAXgene blood RNA tube	Serum<br>Plasma, Buffy coat<br>-
24 h*	6 ml	Clot activator tube	Serum
48 h*	6 ml<br>6 ml<br>2,5 ml	Clot activator tube<br>EDTA-tube<br>PAXgene blood RNA tube	Serum<br>Plasma<br>-
72 h*	6 ml	Clot activator tube	Serum
**In total**	**41 ml**		

*after randomisation

2.2. Sample Collection Kits

The biobank IBBL will provide each site sample collection kits for use in patients enrolled in one or both trials. All the materials will be sent to the site study coordinator at the Intensive Care Unit (ICU), who will in turn distribute as appropriate.

The sample collection kits will include pre-labelled collection tubes, Kit ID labels for sample collection forms, and resource materials for the laboratory (cryovials and labels). Each supplied kit contains all the necessary materials for one patient. Each kit will have four bags, one for each time point. After the bedside sample collection, the laboratory materials in the bag should be sent to the laboratory with the samples.

The kit ID, ########-TT-VAR, on the labels, identifies a specific patient and should be recorded in the patient´s medical record. Unused materials must not be used for another trial patient and after the last timepoint the kit should be discarded.

2.3. Sample Collection Forms

The TTM2/TAME sample collection form as shown in Appendix A should be used, but may be translated and adapted to local routines. **The site study coordinator should print the sample collection form double sided!** Please follow the instructions in the sample collection form carefully. Complete a new sample collection form for each time point and send it with the samples and the materials for sample processing to the local laboratory. The laboratory will retain each sample collection form and the site study coordinator should collect the forms at regular intervals for data entry purposes.

3. SAMPLE PREPARATION AND PROCESSING AT THE SITE LABORATORY

The samples will usually be processed by personnel at the local hospital laboratory, but could be processed by the study site personnel at the ICU if the appropriate training has been given.

3.1 Materials for sample processing

The site laboratory will receive blood samples from the ICU four times/patient, or less if a patient is discontinued from the trial. At each timepoint the samples will be sent with a sample collection form and a bag with sufficient materials for the samples to be processed. Each bag will contain cryovials and labels for the cryovials. Boxes for storage of the frozen cryovials will be provided by IBBL before the site commence recruitment.

The ICU will ensure that the correct labels for the cryovials are provided with the blood samples. **The laboratory should be careful to choose the labels for the correct sample type according to the processing instructions.** The labels include the following information (TT being the TTM2/TAME trials identifier):

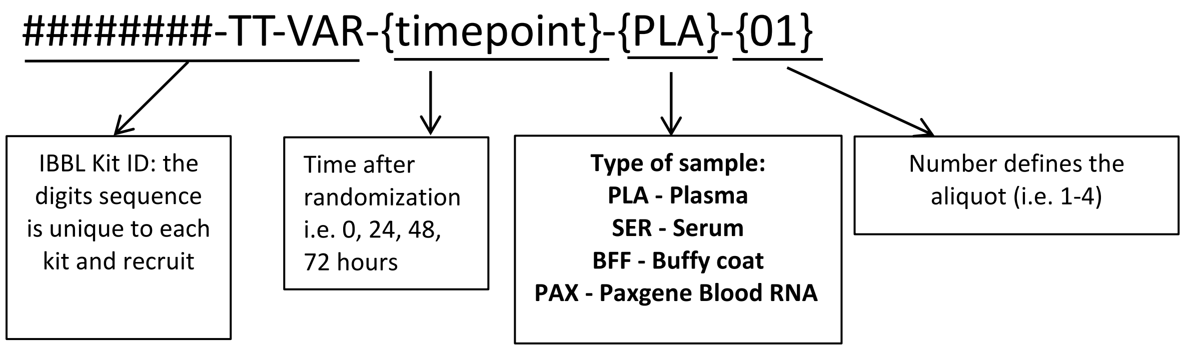

3.2. How to apply the labels

The labels are designed to stick onto clean and dry cryovials at ambient temperature. Use the following method:

- Pull on the support to peel off the label. Be careful not to touch the sticky part of the label.
- Place the long side of the label perpendicular to the cryovial held in the upright direction, as shown in the pictures below. **Both the barcode and the readable text must be visible!**

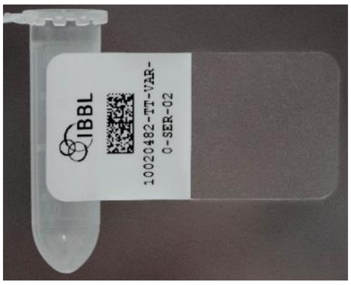

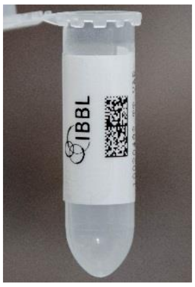

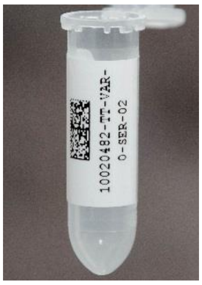

- Start with placing the white part of the label on the tube, and then rolling it firmly around the cryovial to ensure a good contact and adherence. The transparent part of the label will go over the white part as a second layer.
- Be sure that there are no air bubbles between the label and the cryovial.

If the cryovial for some reason is already frozen, remove the frost on the outer wall before the label is applied. The application procedure must be performed within 4 seconds and the cryovials immediately be put back in the freezer.

3.3. Sample processing instructions

Use the cryovials, labels, and sample collection form provided together with the blood collection tubes to process. **The provided labels include the correct kit ID and timepoint, but please be careful to choose the labels for the right sample type (SER, PLA or BFF)!**

Serum (at 0h, 24h, 48h, and 72h)

1. Allow the blood in the clot activator tube to clot for 30 min after sampling at room temperature.
2. Label 4 cryovials with the provided labels "########-TT-VAR-{timepoint}-**SER**-{01-04}"
3. Centrifuge the clot-activator tube at 2000 g for 10 min at room temperature (slow deceleration at the end of centrifugation).
4. Record the time of centrifugation on the sample collection form.
5. Using a disposable pipette, carefully transfer the serum into an appropriate unused plastic tube. Save the tube containing serum and discard the tube with the clot.
6. Homogenize the serum by up and down movements with a pipette.
7. Aliquot the total volume of serum into the 4 labelled cryovials. Split the serum evenly between the cryovials (if possible at least 500 µL/aliquot).
8. **As soon as possible put the labelled cryovials in long term storage at -80°C in the boxes provided.**
9. Record the time of freezing on the sample collection form.

Plasma (at 0 h and 48 h)

1. Centrifuge the EDTA- tube as soon as possible at 2000 g for 10 min at room temperature (slow deceleration at the end).
2. Record the time of centrifugation on the sample collection form.
3. Label 4 cryovials with the provided labels "########-TT-VAR -{timepoint}-**PLA**-{01-04}"

4. Using a disposable pipette, carefully transfer the plasma into an appropriate unused plastic tube.
5. **Applies only to the sample drawn at 0h (not at 48h):** After removing the plasma, transfer the buffy coat (the thin layer beneath the plasma) and a small portion of the red blood cells with a disposable pipette to a cryovial labelled with "########-TT-VAR-0-**BFF**-01". To ensure all the buffy coat is extracted transfer at least 500 µL. Discard the collection tube containing the rest of the blood.
6. Homogenize the plasma obtained at point 4 by up and down movements with a pipette.
7. Aliquot the total volume of plasma into the 4 labelled cryovials. Split the plasma evenly between the 4 cryovials (if possible at least 500 µL/aliquot).
8. **As soon as possible put cryovials in long term storage at -80°C in the provided boxes**
9. Record the time of freezing on the sample collection form.

PAXgene tube (at 0 h and 48 h)

1. Leave the pre-labelled PAXgene tube upright at room temperature for a minimum of 2 hours and a maximum of 24 hours after sample collection.
2. Place the tube in long term storage at -20°C.
3. Record the time of freezing on the sample collection form.

3.4 Sample Storage Log

All the TTM2/TAME samples in storage at the local laboratory should be registered in a sample storage log. The cryovials should be placed in the provided storage boxes in the same order as they are listed in the storage log. The log should preferably be completed as soon as the samples are being put in the freezer, but could be created prior to shipment to the biobank IBBL. The sample storage log as shown in Appendix B, or another log with corresponding information, may be used.

The boxes with the cryovials must be stored at -80°C and the PAXgene tubes at -20°C in freezers at the hospital. Temperature logs should be kept for all the freezers storing samples.

4. SHIPMENT

Samples should be shipped to IBBL in Luxembourg at the end of the trial, or if necessary at regular intervals (not more than every 6 months to avoid unnecessary shipping costs). The shipment will be organized by the sponsor in collaboration with the site study coordinator. A courier customer code will be given, allowing for the shipment costs to be paid for by the sponsor. Detailed instructions on shipping will be part of a separate form.

5. CONTACT

Contact for laboratory/biomarker questions:

TTM2: helena.levin@med.lu.se or ttm2@ttm2trial.org
TAME: ciara.fahey@ucd.ie

TTM2/TAME SAMPLE COLLECTION FORM

Blood Collection at the Site

Patient study number:
**Kit ID label** (########-TT-VAR):

Contact at the ICU:
Name: <complete>
Tel: <complete>
Email: <complete>

Sampling should be performed as close as possible to the designated time point. Samples may be drawn +/- 6 hours from the ideal time point to allow correct laboratory processing.

1. **Use a sampling kit containing the necessary materials for one patient. Each kit includes Kit ID labels for forms and 4 bags with blood collection tubes and laboratory materials.**
2. **Fill out a new sample collection form for each time point. Record the correct patient study number and place a Kit ID label, ########-TT-VAR, in the red square above!**

   Sampling time point (hours after randomisation):

   0 h ☐ 24 h ☐ 48 h ☐ 72 h ☐

   Sampling date and time:

   _ _ _ _ _ 2 0 _ _   _ _ : _ _
   D D M M M Y Y Y Y   H H : M M

   Record month with 3 letters and time in 24-hour format, e.g. 15DEC2017 15:30

   Sampling performed by (name/initials):

   ……………………………………………………………

3. **Choose the bag marked with the correct time point and draw the samples with the pre-labelled tubes in the following order. Keep the PAXgene tube upright to prevent backflow.**

   At 0 h and 48 h:
   1 x Clot activator tube (red cap)
   1 x EDTA tube (purple cap)
   1 x PAXgene tube

   At 24 h and 72 h:
   1 x Clot activator tube (red cap)

4. **Invert the clot activator tube (red) 5-6 times. Invert the EDTA tube (purple) and PAX tube 8-10 times.**
5. **Send this form and the remaining materials in the bag (cryovials and labels) to the laboratory with the samples**.
6. **At the timepoint 0 h, record the patient´s kit ID, ########-TT-VAR, in the medical record.**
   The kit ID is the same for all time points.

PLEASE BE CAREFUL TO COMPLETE THE RED SQUARE AND TO SEND MATERIALS TO THE LABORATORY!

After the last time point (72h), discard any unused material. The kit must not be used for another patient.

TTM2/TAME SAMPLE COLLECTION FORM

Sample Processing at the Laboratory

PLEASE VERIFY THAT THE RED SQUARE ON THE BACK OF THIS FORM HAS BEEN COMPLETED.
If the patient study number or the Kit ID is missing, please contact the ICU!

Handle the samples according to the provided instructions and complete the information below.

Record date and time in the format DDMMMYYYY HH:MM e.g. 15DEC2017 15:30

Save this form! The forms will be collected at regular intervals by the site coordinator and the data will be entered in the eCRF at the ICU.

- **Serum** (at 0h, 24h, 48h, and 72h):

Date and time of centrifugation:

_ _ _ _ _ 2 0 _ _ _ _ _ _
D D M M M Y Y Y Y H H : M M

Date and time placed in freezer:

_ _ _ _ _ 2 0 _ _ _ _ _ _
D D M M M Y Y Y Y H H : M M

- **Plasma** (at 0 h and 48 h):

Date and time of centrifugation:

_ _ _ _ _ 2 0 _ _ _ _ _ _
D D M M M Y Y Y Y H H : M M

Date and time placed in freezer:

_ _ _ _ _ 2 0 _ _ _ _ _ _
D D M M M Y Y Y Y H H : M M

- **PAXgene tube** (at 0 h and 48 h):

Date and time placed in freezer:

_ _ _ _ _ 2 0 _ _ _ _ _ _
D D M M M Y Y Y Y H H : M M

Samples handled by (name/initials):

……………………………………………………………

If any deviations occurred, please specify:

TTM2/TAME SAMPLE STORAGE LOG

**Patient´s Kit ID**<br>########-TT-VAR<br>(first 8 digits on the label)	**Time Point**	**Sample Type**	**Number of Vials in Storage**	**Storage in Box No(s)**	**Date and Initials**
	0	Serum			
	0	Plasma			
	0	Buffy coat			
	0	PAXgene			
	24	Serum			
	48	Serum			
	48	Plasma			
	48	PAXgene			
	72	Serum			
	0	Serum			
	0	Plasma			
	0	Buffy coat			
	0	PAXgene			
	24	Serum			
	48	Serum			
	48	Plasma			
	48	PAXgene			
	72	Serum			
	0	Serum			
	0	Plasma			
	0	Buffy coat			
	0	PAXgene			
	24	Serum			
	48	Serum			
	48	Plasma			
	48	PAXgene			
	72	Serum			

Page no: _______ of ________
